# Draft genome sequences of three *Planktothrix rubescens* strains cultivated from a eutrophic lake in Norwalk, Ohio (USA)

**DOI:** 10.1128/mra.00708-24

**Published:** 2024-09-09

**Authors:** Ryan S. Wagner, George S. Bullerjahn

**Affiliations:** 1 Department of Biological Sciences, Bowling Green State University, Bowling Green, Ohio, USA; 2 Great Lakes Center for Fresh Waters and Human Health, Bowling Green State University, Bowling Green, Ohio, USA; SUNY College of Environmental Science and Forestry, Syracuse, New York, USA

**Keywords:** cyanobacteria, cyanotoxin, freshwater, harmful algal blooms

## Abstract

Draft genomes were generated for three filamentous toxin-producing cyanobacterial strains cultivated from aquatic sources in Ohio sequenced by NovaSeq S4. Here, we report the classification and genome statistics of *Planktothrix rubescens* PR221, PR222, and PR223.

## ANNOUNCEMENT

*Planktothrix* spp. are filamentous cyanobacteria commonly found in eutrophic waters, and many species produce toxins (1). Two of the most common taxa are *Planktothrix agardhii* and *Planktothrix rubescens*, which occupy different niches in freshwaters. *P. rubescens* is found in the metalimnion while *P. agardhii* the epilimnion (2, 3). Both taxa contain gas vesicles allowing them to migrate through the water column to their optimal light intensity (4). Because *P. rubescens* has been shown to be toxic (1), understanding under what conditions *P. rubescens* grows will be valuable for protecting water supplies (5).

Skinn Lake is a former rock quarry located in Norwalk, Ohio (41.22 N, −82.63 W), with a depth of 12 m. The lake sees annual winter *Planktothrix rubescens* blooms, which become visible during mixing events. Grab samples were taken from the shoreline using a bottle on a pole, placed on ice, and brought back for culturing of dominant taxa. Preliminary identification used microscopy to identify species. After identification and cultivation from three separate grab samples, preparation of xenic, unialgal cultures was done from each sample using single filament picking with a glass pipette in liquid JM media (6) and incubated at 12°C at 10 µmol quanta m^−2^ s^−1^ with a light dark cycle of 14–10 h. After reculturing of selected single filaments, DNA was extracted by filtering biomass through a 0.22-µm Sterivex filter cartridge. Filters were frozen at −80°C before DNA extraction. Sterivex cartridges were then cut open, and the filters were removed for extraction with the DNeasy PowerWater Kit (Qiagen, Germantown, MD) according to the manufacturer’s instructions. DNA libraries were prepared and sequenced by the University of Minnesota Genomics Center (Minneapolis, MN). Libraries were created using the Illumina NexteraXT Kit and sequenced on a NovaSeq S4 to generate 150-bp paired-end reads yielding 307,960,113, 285,974,114, and 278,521,261 total sequences for PR221, PR222, and PR223 respectively (Table 1).

**TABLE 1:** Genome statistics on Skinn Lake *P. rubescens*

Characteristic	*Planktothrix rubescens*		
	PR221	PR222	PR223
Genome length	5,534,738	5,533,187	5,567,603
No. of contigs	134	125	140
GC content (%)	39.42	39.38	39.43
Completeness (%)	99.34	99.56	99.78
Contamination (%)	2.29	2.29	2.29
N_50_ (bp)	66,822	66,509	68,205
FastANI reference	GCF_900009275.1	GCF_900009275.1	GCF_900009275.1
FastANI organism name	*Planktothrix rubescens* NIVA-CYA 18	*Planktothrix rubescens* NIVA-CYA 18	*Planktothrix rubescens* NIVA-CYA 18
FastANI ANI (%)	98.23	98.27	98.29
Assembly depth of coverage	392.846	414.208	387.251
NCBI PGAP			
CDS	4,971	4,988	5,041
tRNAs	38	38	38
rRNAs	2	3	3
ncRNAs	4	4	4
Totals	5,015	5,033	5,086

Reads were uploaded to the DOE Systems Biology Knowledgebase (KBase) v2.7.11 (7) for further analysis, and all parameters were run using default settings. Reads were then trimmed to remove low-quality base calls, and adapters were clipped using Trimmomatic v0.36 (8). After trimming, the quality of reads was assessed using FastQC v0.11.9 (9). Samples were assembled with a minimum of 2,000 bp using metaSPAdes v3.15.3 (10). MaxBin2 v2.2.4 (11), MetaBAT2 v1.7 (12), and CONCOCT v1.1 (13) were used to bin assemblies into contigs using default settings. Contigs were optimized using the DAS-tool v1.1.2 (14) and quality assessed using CheckM v1.0.18 (15). Next, bins were filtered for completeness (90%) and contamination (≤5%) with CheckM. Extracted bins were run through QUAST v4.4 (16) for final quality assessment. Taxonomy was assigned using average nucleotide identity against the publicly available reference genome, *Planktothrix rubescens* NIVA-CYA 18 with FASTANI v0.1.3 (17) and through GTDB-Tk v2.3.2 (18). Genomes were submitted to NCBI for the Prokaryotic Genome Annotation Pipeline (PGAP) v6.7 (19).

## Data Availability

The metagenome-assembled genomes have been deposited in GenBank under the accession numbers JAYWIL000000000, JAYWIM000000000, and JAYWIN000000000 for PR221, PR222, and PR223, respectively. The raw sequence files are available as sequence read archives under SRR27742818, SRR27742817, and SRR27742816. The BioProject can be found under the reference PRJNA1050598.
